# Diagnostic Utility of Urinary Amylase in Acute Pancreatitis: A Systematic Review

**DOI:** 10.7759/cureus.99583

**Published:** 2025-12-18

**Authors:** Ubaid Ullah, Syed Saad Ali Chishti, Asim Junaid, Marria Sarwar

**Affiliations:** 1 Vascular Surgery, Beaumont Hospital, Dublin, IRL; 2 Colorectal Surgery, Beaumont Hospital, Dublin, IRL; 3 General Surgery, University Hospitals Birmingham NHS Foundation Trust, Birmingham, GBR; 4 General Surgery, Shaheed Zulfiqar Ali Bhutto Medical University, Islamabad, PAK

**Keywords:** acute pancreatitis, biomarkers, diagnostic accuracy, systematic review, urinary amylase, urinary trypsinogen-2

## Abstract

Background: Diagnosis of acute pancreatitis (AP) is primarily based on serum amylase and lipase levels, which may normalize rapidly or remain within normal limits in early or late stages. Urinary amylase, with a longer detection window and easier sample collection, may offer a more sensitive diagnostic option, though evidence remains inconsistent.

Objective: This study aimed to systematically review published studies evaluating the diagnostic accuracy of urinary amylase in AP.

Methods: A comprehensive search was conducted across PubMed, Embase, Scopus, Web of Science, Cochrane Library, and Google Scholar for studies published from January 2010 to May 2025. Eligible studies included observational, prospective, or diagnostic accuracy designs assessing urinary amylase in clinically suspected or confirmed AP. Data on sensitivity, specificity, predictive values, and area under the curve (AUC) were extracted. Study quality was assessed using the QUADAS-2 tool.

Results: Thirteen studies (sample sizes 51-458) met the inclusion criteria, evaluating urinary amylase and urinary trypsinogen-2 in AP. Reported sensitivities ranged from 62.9% to 100% and specificities from 42.9% to 100%. Comparative analyses demonstrated that urinary biomarkers often performed as well as or better than serum amylase and lipase, with AUC values reaching up to 1.0. Several studies highlighted the high negative predictive value (up to 98.3%), suggesting strong utility for excluding AP. Urinary testing also provides practical advantages, including being non-invasive, rapid, and repeatable, which make it especially suitable for emergency and resource-limited settings.

Conclusion: Urinary amylase and urinary trypsinogen-2 are sensitive, reliable, and clinically useful biomarkers for diagnosing AP. Their diagnostic performance equals or surpasses conventional serum markers, supporting their integration into diagnostic pathways as first-line or adjunctive tests. Standardized protocols and multicenter validation studies are recommended to optimize their clinical application.

## Introduction and background

Acute pancreatitis (AP) is a frequent gastrointestinal emergency, which is characterized by the abrupt development of severe abdominal pain, frequently followed by nausea and vomiting, and may occur as a mild self-limited disease or a life-threatening one [1]. The need to prevent morbidity and mortality is to ensure that appropriate management is achieved through early diagnosis. Traditionally, biochemical markers that have been extensively used to diagnose AP include serum amylase and lipase. There are a number of limitations to these tests, though, such as low half-life, lack of specificity, and varying sensitivities with the time of presentation [2,3]. On the first presentation, about 20-30% of AP patients can have normal serum amylase levels, especially those who have a late-onset or mild disease, which underlines the necessity to use other diagnostic methods [1,4].

Even though serum amylase has been traditionally regarded as a typical diagnostic indicator of AP, its diagnostic accuracy has been doubted. Gomez et al. conducted a retrospective study and discovered that serum lipase had a better sensitivity (96.6%) and specificity (99.4%) than amylase (78.6% and 99.1%, respectively). These observations demonstrate the drawbacks of serum amylase and support investigating other markers like urinary amylase to diagnose the condition better [5]. A study critically examined the diagnostic utility of pancreatic enzymes and declared that no single biochemical test can meet all the aspects of diagnosing and assessing the severity of AP. Serum amylase and lipase are the most commonly used, but due to the lack of specificity and time-related variability, they have low diagnostic accuracy. The authors reported that urinary markers such as urinary trypsinogen activation peptides can be viable alternative non-invasive methods for the early diagnosis and severity forecasting of AP [6].

Urinary amylase is an alternative biomarker with potential to be used instead of serum amylase because it can be raised longer and there is a possibility of collection without any invasion [2]. Several studies have assessed the use of urinary amylase as a diagnostic test in AP with inconsistent sensitivity and specificity in different groups of patients.

Likewise, a prospective study conducted in a tertiary care hospital discovered that the level of urinary amylase was significantly greater than that of serum amylase, with a mean of 501 U/L and 311 U/L, respectively, thus demonstrating its diagnostic value. The paper has highlighted the fact that urinary amylase may be used to detect AP in situations in which serum markers may be inaccurately normal, specifically in patients who develop pancreatitis late or with mild pancreatitis [7]. These were supported by authors who showed that urinary amylase was more sensitive and specific than serum amylase or lipase when measured at admission, showing that it can be used as a first-line diagnostic tool [8].

The clinical usefulness of urinary amylase has also been confirmed by prospective case-control studies. Researchers conducted a comparative study between urinary amylase, serum amylase, and serum lipase in 100 patients and reported a 97.25 and 91.47 urinary amylase sensitivity and specificity, respectively, which are higher and comparable to serum markers [9]. The authors of one study introduced a novel urinary strip for the detection of urinary amylase. They reported a sensitivity of 69% and a specificity of 97%. This provides reasonable and rapid point-of-care urinary amylase levels in the emergency department [10].** **According to a study, urinary amylase also rendered similar diagnostic accuracy as serum amylase and lipase and added the benefits of convenience and non-invasive sampling [11].

Comparison between urinary and serum amylase as a diagnostic measure has been addressed in several local researches. Urinary amylase was more sensitive to all the levels of AP severity, especially in moderate disease (77.27%) and patients who reported the disease late (after four days of symptom onset) (81.81%) [2]. One of the research articles emphasized that, if higher diagnostic threshold values were set for serum amylase and lipase, the specificity of the test increases but its sensitivity decreases, while urinary amylase retains a good balance between the two.** **These results highlight urinary amylase as a potentially more useful biomarker of AP, especially in emergency and delayed presentations [3].

Balaji et al. used a prospective comparative design in a tertiary care hospital to compare the diagnostic capacity of a rapid urinary trypsinogen-2 strip test with the conventional serum amylase and lipase tests in unique cases of AP. The researchers recruited 52 patients who came with acute abdominal pain, of whom 45 patients were diagnosed with AP. The urinary trypsinogen-2 dipstick test had a high sensitivity of 97.78%, a specificity of 71.43%, and a diagnostic accuracy of 94.23%. The authors also determined that the rapid urinary trypsinogen-2 is a fast, consistent, and non-invasive diagnostic measure that can be a suitable substitute for the usual biochemical markers to help in the initial diagnosis of AP [12].

These results are also supported by large systematic evaluations. The Cochrane review carried out research that summarized the results of 10 studies (with more than 5,000 participants) and found that serum amylase, serum lipase, and urinary trypsinogen-2 exhibited the same sensitivity (0.72-0.79) and specificity (0.89-0.93), but still about a quarter of patients with AP were not diagnosed by these measurements. The review proposed low thresholds to be further investigated, which further supports the need to strengthen query biomarkers like urinary amylase [1].

Subsequent clinical observations also support the application of urinary amylase. A single-center study in the country of Ireland indicated that the patients with AP had mean urinary amylase levels of 5,034 U/L that were significantly higher than serum amylase and the association was very high (strong), with a specificity of 97% [4]. Correspondingly, one study reported that not only did urinary amylase increase the level of diagnostic accuracy but also presented better disease monitoring because of its sustained elevation in comparison with serum markers [8,13].

On the whole, these studies all bring out urinary amylase as a non-invasive, sensitive, and specific biomarker of AP. Urinary amylase has demonstrated benefits compared to serum amylase and lipase in terms of early detection, delay of presentation, and possible prognosis evaluation. Although there was variability in the reported sensitivity and specificity, evidence has been found that suggests it can be incorporated into clinical practice as an adjunctive test or primary diagnostic test. The literature review highlights why a systematic review should be carried out to aid in the incorporation of these results to formulate pooled estimates of diagnostic accuracy and clinical recommendations to use urinary amylase in AP.

## Review

Methods

Study Design

It is a systematic review that took place in compliance with the requirements of the Preferred Reporting Items for Systematic Reviews and Meta-Analyses (PRISMA) 2020 [14]. These were aimed at determining the diagnostic value of urinary amylase in AP relative to serum amylase, serum lipase, and other diagnostic biomarkers.

Search Strategy

An electronic search was conducted to find all the studies that were related and published between January 2010 and May 2025. The databases PubMed, Embase (Elsevier), Web of Science, Scopus, and Cochrane Library were examined to search for literature. Grey literature was searched using Google Scholar and some freely accessible resources like the World Health Organization (WHO) Institutional Repository for Information Sharing (IRIS), SciSpace, and ProQuest Dissertations and Theses Global.

The most recent search update was done in the year 2025. Articles and reviews included in the reference lists were also manually screened in order to get other eligible studies. Peer-reviewed articles and books were included, and only those published in English were used due to limited resources and translation constraints. 

Search Terms and Boolean Operators

The search strategy was a combination of the Medical Subject Headings (MeSH) and free-text terms concerning urinary amylase and AP. Optimization of search precision and sensitivity was done using Boolean operators (AND, OR) and truncation symbols [15].

To be as sensitive and as precise as possible, the electronic search was conducted in a combination of MeSH terms and free-text keywords. The search used in PubMed was as follows: Urinary Amylase [MeSH] or urinary amylase, and Acute Pancreatitis [MeSH] or acute pancreatic inflammation, and acute pancreatic disease. The search logic was organized with the help of Boolean operators (AND, OR) to be able to retrieve as many relevant articles as possible. Other databases, such as Embase, Web of Science, Scopus, and Cochrane Library, were altered to use similar tailored search strategies in order to find all the relevant studies on urinary biomarkers in AP.

Inclusion Criteria

The studies were eligible if they satisfied the following criteria. The Population included adults or childhood patients who had a clinically suspected or confirmed AP. The Index Test was evaluated by using urinary amylase measured by dipstick, quantitative, or semi-quantitative tests. Studies were required to include a comparator, defined as diagnostic criteria of reference such as serum amylase, serum lipase, or image verification, i.e., CT, MRI, or ultrasound. At least one outcome in terms of diagnostic performance is reported: sensitivity, specificity, positive predictive value (PPV), negative predictive value (NPV), or diagnostic accuracy. Moreover, the study designs included observational (prospective or retrospective), diagnostic accuracy, or cross-sectional studies. At last, only English peer-reviewed articles published since 2010 were included.

Exclusion Criteria

The studies were eliminated if they concentrated on non-humans or animal models. One more criterion for exclusion was that only chronic pancreatitis or other disorders involving the pancreas were considered. In addition, case reports, reviews, editorials, or conference abstracts with inadequate data were excluded. The most important and considerable point was that those studies were excluded if they failed to consider urinary amylase as a first- or second-line diagnostic parameter.

Selection and Screening Process

**Figure 1:**
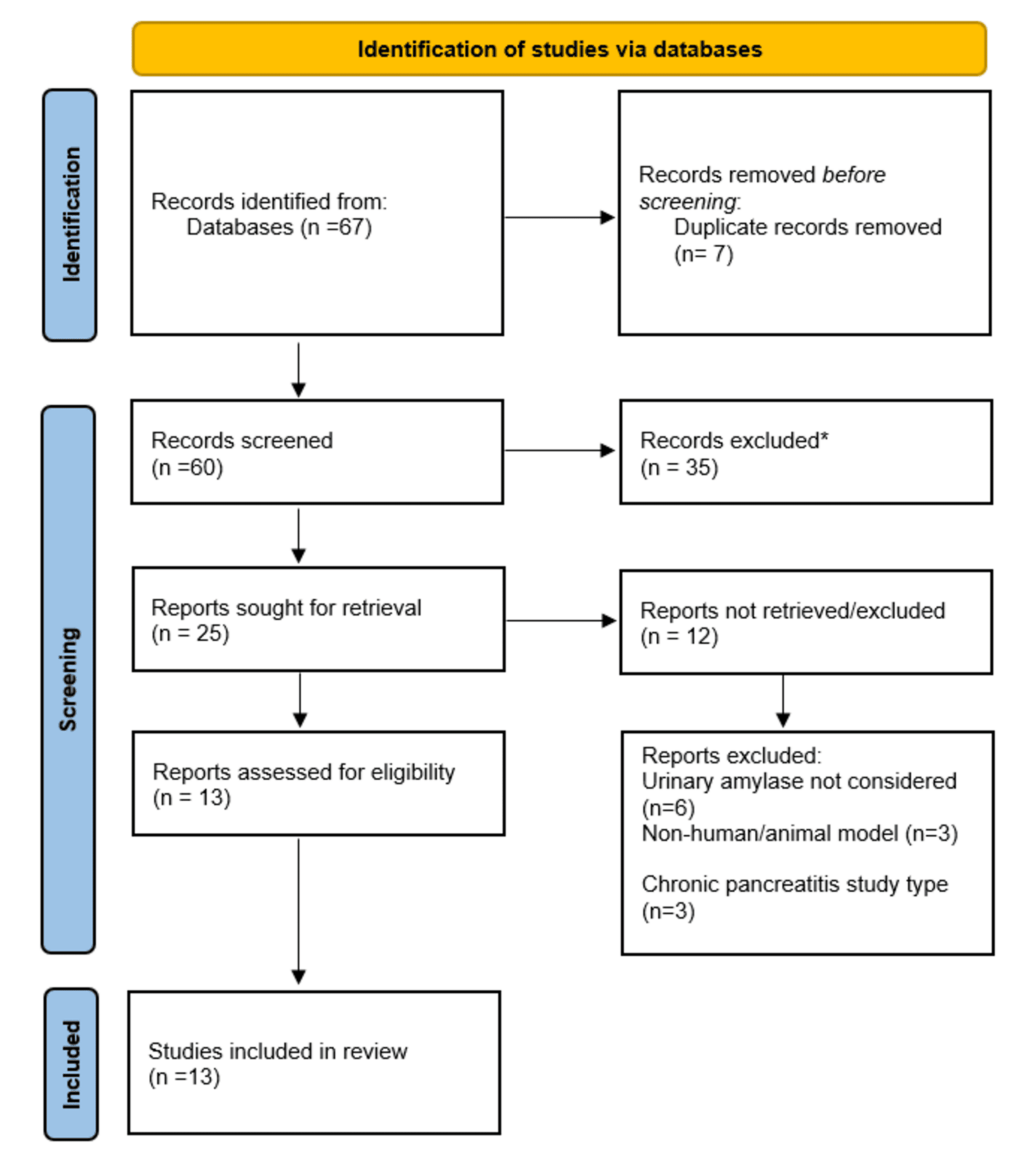
PRISMA flow diagram illustrating the study selection process This diagram depicts the flow of records through the phases of the systematic review: Identification, Screening, and Inclusion. *Studies were excluded after screening titles and abstracts due to irrelevance and failure to meet the inclusion criteria. PRISMA: Preferred Reporting Items for Systematic Reviews and Meta-Analyses

All of the retrieved records were imported into EndNote 21 (Clarivate, London, United Kingdom), where they would be managed as references and duplicates would be removed [16]. Titles and abstracts were later screened to remove irrelevant entries, after which full-text assessment was done to ascertain eligibility. Where the discrepancies occurred, they were addressed by either discussing or consulting a third reviewer. The whole process of selection was recorded according to the PRISMA 2020 flow diagram (Figure 1), and further numbers of identified, screened, excluded (with reasons), and included studies in the final synthesis were documented [14].

Data Extraction

A standardized form was used to ensure consistency across studies. Information recorded included the author and year of publication, country and study setting, study design, and sample size. Details of the index diagnostic tests (such as urinary amylase or urinary trypsinogen-2) and comparator tests (including serum amylase, serum lipase, or imaging modalities) were collected, along with any reported cut-off or threshold values. Diagnostic performance metrics, including sensitivity, specificity, PPV, NPV, and overall diagnostic accuracy, were extracted where available. The reference standard used to confirm the diagnosis, such as CT findings or the Revised Atlanta Criteria, was also documented. Additional notes were recorded to capture any relevant methodological considerations, population characteristics, or contextual factors affecting interpretation.

Quality Assessment

The quality of the methodology and risk of bias of each study included was assessed with the use of the QUADAS-2 tool, which evaluated four domains: patient selection, index test, reference standard, and flow and timing. Each study was carefully reviewed across these domains to determine potential sources of bias and applicability concerns. A narrative synthesis was, first of all, performed considering the expected heterogeneity in the study design, cut-off values, and diagnostic criteria [17].

In cases where three or more studies provided similar results, it was intended to pool the performance of the diagnostic (in terms of sensitivity, specificity, and area under the curve (AUC)) using a random-effects bivariate model in Stata 17 (StataCorp LLC, College Station, Texas, United States) [18].

Ethical Considerations

As this review used the data from already published studies, no ethical approval was necessary. Each of the procedures was carried out using the rules of transparency, reproducibility, and scholarly integrity.

Results

A total of 67 articles were first identified using a systematic search, of which 13 articles met the predefined inclusion criteria and were incorporated in this review. These papers were published between 2010 and 2025 and had sample sizes between 51 and 458 participants in India, Japan, Pakistan, Iran, and Ireland. Most of the studies used the prospective observational design with three cross-sectional studies and one randomized clinical trial, which guaranteed a strong evaluation of urinary amylase as a diagnostic tool. The studies incorporated had urinary amylase or urinary trypsinogen-2 as the index test, and serum amylase, serum lipase, or imaging tests (CT, MRI, ultrasound) served as the reference tests to validate AP.

The nature of the included studies is in Table 1. In the 13 studies, urinary trypsinogen-2 dipstick tests were used in a number of Indian and Japanese studies [19-21], and they showed that they could be used as a rapid point-of-care diagnostic tool. The sample sizes were very diverse, including tertiary hospital cohorts as well as multicenter population studies. The majority of the research involved adults, but a few of them tested child cases as well. The urinary amylase measurement methods were involved in quantitative, semi-quantitative, and serial monitoring methods, which gave information about the early diagnosis as well as the monitoring of the disease progression. In the studies, like Meena et al., the urinary trypsinogen-2 concentrations were associated with disease severity, and Mogekar et al. showed better diagnostic capabilities of urinary amylase than serum amylase, which is why this diagnostic method could be used in clinical practice [21,7].

**Table 1:** Characteristics of the included studies (n=13) This table summarizes the characteristics of the 13 included studies, including author and year, country, study design, sample size, index tests, comparator(s), reference standards, and key findings. TAP: trypsinogen activation peptide; PEP: post-endoscopic retrograde cholangiopancreatography pancreatitis; CRP: C-reactive protein; UTT: urinary trypsinogen-2 dipstick test; AUC: area under the curve; AP: acute pancreatitis; NA: not available

Author (year)	Country	Study design	Sample size	Index test(s)	Comparator(s)	Reference standard	Key findings
Yasuda et al. (2019) [19]	Japan	Multicenter prospective	94	Urinary trypsinogen-2, TAP	Serum amylase, lipase	CT, Ministry of Health criteria	Urinary trypsinogen-2 dipstick showed 73.1% sensitivity; useful in diagnosis and grading
Yewale et al. (2022) [20]	India	Prospective analytical	79	Urinary trypsinogen-2 dipstick	Serum lipase	Clinical + biochemical diagnosis	Sensitivity 66.7% and specificity 92.1%; helpful in ruling out PEP
Mogekar et al. (2024) [7]	India	Cross-sectional	60	Urinary amylase	Serum amylase, lipase	Clinical + radiological diagnosis	Urinary amylase (501 U/L) more reliable than serum amylase; higher diagnostic accuracy
Meena et al. (2024) [21]	India	Prospective	60	Urinary trypsinogen-2	Serum amylase, lipase, CRP	Revised Atlanta classification	Urinary trypsinogen-2 correlated with severity; sensitivity 92.3% and specificity 42.9%
Raja et al. (2019) [22]	India	Prospective observational	80	Urinary trypsinogen-2 dipstick	Serum amylase, lipase	Clinical + imaging	UTT sensitivity 92.7% and specificity 98.5%; rapid, reliable point-of-care screening
Kumar et al. (2021) [23]	India	Observational	100	Urinary trypsinogen-2 test strip	Serum amylase, lipase	Clinical + biochemical criteria	Urinary trypsinogen-2 more sensitive (96.1%) than serum lipase (90.2%) and serum amylase (84.3%)
Judal et al. (2022) [9]	India	Prospective case-control	100	Urinary amylase	Serum amylase, lipase	Clinical + imaging	Urinary amylase sensitivity 97.25% and specificity 91.47%; AUC=0.934
Akula et al. (2025) [13]	India	Cross-sectional	55	Urinary amylase (serial)	Serum amylase, lipase	Revised Atlanta classification	Urinary amylase had best AUC=0.878, sensitivity 95%, and specificity 92%
Shahid et al. (2022) [8]	Pakistan	Randomized clinical control	180	Urinary amylase	Serum amylase, lipase	Clinical + radiological	Urinary amylase significant (p<0.05); AUC=1.0; superior diagnostic discrimination
Mohammed et al. (2023) [4]	Ireland	Observational	190	Urinary amylase	Serum amylase	CT abdomen	Mean urinary amylase 5034 U/L vs. serum 233 U/L; specificity ~97%
Gupta et al. (2017) [2]	India	Comparative observational	51	Urinary amylase	Serum amylase	Contrast-enhanced CT	Urinary amylase sensitivity higher across all stages (up to 81.8% after eight days)
Esmaili et al. (2017) [3]	Iran	Descriptive-analytical	458	Serum amylase, lipase	NA	Clinical + radiological	Amylase sensitivity 79.2% and specificity 69%; lipase sensitivity 80% and specificity 69%; both useful but limited specificity
Karichery and Haridas (2023) [11]	India	Prospective case-control	200	Urinary amylase	Serum amylase, lipase	Clinical + radiological	Urinary amylase showed diagnostic accuracy comparable to serum enzymes; useful in mild/late AP

Table 2 shows the metrics of diagnostic accuracy of the utilized studies. In the literature, the sensitivity of urinary amylase/trypsinogen-2 was found to range as high as 100% to 62.9% with the highest reliability in diagnosing AP, especially in mild or early disease. Specificity ranged between 42.9% and 100%, with lesser specificity in the studies that dealt with the assessment of severity due to the effect of inflammation or comorbid conditions. It is worth noting that Judal et al. achieved a sensitivity of 97.25%, a specificity of 91.47%, and an AUC of 0.934, which means that it has an excellent discriminative ability [9]. Equally, Akula et al. achieved a sensitivity of 95% and a specificity of 92% with an AUC of 0.878, which supports urinary amylase as a useful predictor of different patient groups [13]. It is also beneficial, as evidenced by the high negative predictive values of 98.3% by Yewale et al., which helps to eliminate AP, eliminating the need to waste resources [20]. These results indicate that urinary amylase can be used as a non-invasive and rapid diagnostic test to supplement the conventional serum markers and imaging, especially in emergency or resource-constrained situations.

**Table 2:** Diagnostic accuracy metrics of the included studies This table summarizes the diagnostic accuracy metrics of the included studies, including sensitivity, specificity, PPV, NPV, AUC, and any relevant remarks. All values were extracted from the original studies. PPV: positive predictive value; NPV: negative predictive value; AUC: area under the curve; ERCP: endoscopic retrograde cholangiopancreatography pancreatitis; AP: acute pancreatitis; UTT: urinary trypsinogen-2 dipstick test; NA: marker not reported

Study	Sensitivity (%)	Specificity (%)	PPV (%)	NPV (%)	AUC	Remarks
Yasuda et al. (2019) [19]	73.1	62.5	NA	NA	0.704	Urinary trypsinogen-2 correlated with inflammation grade
Yewale et al. (2022) [20]	66.7	92.1	25	98.3	0.91 (approx.)	Useful for early post-ERCP screening
Mogekar et al. (2024) [7]	NA	NA	NA	NA	NA	Urinary amylase more reliable than serum amylase
Meena et al. (2024) [21]	92.3	42.9	75	75	NA	Indicates the severity of AP
Raja et al. (2019) [22]	92.7	98.5	NA	NA	NA	UTT rapid test accurate and quick
Kumar et al. (2021) [23]	96.1	82.6	NA	NA	NA	High NPV=90.5%
Judal et al. (2022) [9]	97.25	91.47	NA	NA	0.934	Comparable to lipase AUC=0.945
Akula et al. (2025) [13]	95	92	NA	NA	0.878	Urinary amylase superior diagnostic marker
Shahid et al. (2022) [8]	NA	NA	NA	NA	1.00	Highest diagnostic accuracy reported
Mohammed et al. (2023) [4]	NA	97	NA	NA	NA	Urinary amylase strongly associated with CT-confirmed AP
Gupta et al. (2017) [2]	62.9	81.8	NA	NA	NA	Higher sensitivity at late presentation
Esmaili et al. (2017) [3]	79.2	69	43	92	NA	Useful at low cut-off but poor PPV
Karichery and Haridas (2023) [11]	89	97.5	97	89	0.93	Urinary amylase equal diagnostic accuracy to serum tests

Table 3 shows the quality evaluation of the included studies with the help of the QUADAS-2 tool [17]. The majority of the studies had a low risk of bias in patient selection, index test, reference standard, and flow/timing areas. Exceptions were Meena et al. with a moderate risk, because it had limited blinding when interpreting index tests, and Mohammed et al. with an unclear risk, because it had an ambiguous index test domain [21,4]. Altogether, the quality of methodology of the studies used outlines the validity of urinary amylase as a diagnostic marker and provides a strong confidence in the provided sensitivity and specificity measures. The good study designs will make the clinical inferences on the issue of early detection and prediction of severity consistent and generalizable to the real world.

**Table 3:** Quality assessment of the included studies using QUADAS-2 This table presents the quality assessment of the included studies using the QUADAS-2 tool. Domains assessed include patient selection, index test, reference standard, flow and timing, and the overall risk of bias for each study. "Low", "High", or "Unclear" indicates the level of risk in each domain as per the QUADAS-2 guidelines.

Study	Patient selection	Index test	Reference standard	Flow and timing	Overall risk of bias
Yasuda et al. (2019) [19]	Low	Low	Low	Low	Low
Yewale et al. (2022) [20]	Low	Low	Low	Unclear	Low
Mogekar et al. (2024) [7]	Low	Low	Low	Low	Low
Meena et al. (2024) [21]	Low	High (limited blinding)	Low	Unclear	Moderate
Raja et al. (2019) [22]	Low	Low	Low	Low	Low
Kumar et al. (2021) [23]	Low	Low	Low	Low	Low
Judal et al. (2022) [9]	Low	Low	Low	Low	Low
Akula et al. (2025) [13]	Low	Low	Low	Low	Low
Shahid et al. (2022) [8]	Low	Low	Low	Low	Low
Mohammed et al. (2023) [4]	Low	Unclear	Low	Low	Low
Gupta et al. (2017) [2]	Low	Low	Low	Unclear	Low
Esmaili et al. (2017) [3]	Low	Low	Low	Unclear	Low
Karichery and Haridas (2023) [11]	Low	Low	Low	Low	Low

Table 4 presents a comparison of the diagnostic performance of urinary amylase, serum amylase, and serum lipase. Urinary amylase also exhibited the same or higher diagnostic efficacy compared to serum markers. Sensitivity and specificity (97.3% and 91.5%), as reported by Judal et al., were close to the serum lipase specificity (96.5% and AUC 0.945) [9]. Urinary amylase was found to be better than serum amylase (AUC 0.878 vs. 0.532), and it was more characteristic of discriminatory power [13]. According to Shahid et al., there was a perfect diagnostic discrimination (AUC=1.0) that indicated the clinical reliability of urinary amylase in the diagnosis and stratification of the severity of the disease [8]. Similar performance with serum markers was shown by Karichery and Haridas, making it possible to state that urinary amylase can be used as a non-invasive diagnostic test at the first line, in particular, when the situation in the emergency room is associated with the need to make a decision quickly [11]. These findings together would argue in favor of the use of urinary amylase testing as part of routine diagnostic procedures for the symptoms of AP, providing an urgent and dependable test that does not require invasive interventions.

**Table 4:** Comparative diagnostic performance in studies evaluating all three markers This table compares the diagnostic performance of urinary amylase, serum amylase, and serum lipase across studies evaluating all three markers. Sensitivity and specificity are reported for each marker, along with a summary conclusion for each study. Se: sensitivity; Sp: specificity; AUC: area under the curve; NA: data not reported

Study	Urinary amylase (Se/Sp)	Serum amylase (Se/Sp)	Serum lipase (Se/Sp)	Summary conclusion
Mogekar et al. (2024) [7]	NA	NA	NA	Urinary amylase had a higher mean and diagnostic relevance than serum amylase
Judal et al. (2022) [9]	97.3/91.5	100/NA	NA/96.5	Urinary amylase almost as accurate as lipase (AUC=0.934 vs. 0.945)
Akula et al. (2025) [13]	95/92	NA	NA	Urinary amylase superior (AUC=0.878) than serum amylase (AUC=0.532)
Shahid et al. (2022) [8]	100/100 (AUC=1.0)	NA	NA	Urinary amylase best diagnostic discrimination and severity correlation
Karichery and Haridas (2023) [11]	89/97.5 (AUC=0.93)	96/97.5 (AUC=0.97)	100/97.5 (AUC=0.99)	Urinary amylase highly sensitive, reliable, and suitable as a first-line diagnostic test

The accumulated data, clinically, shows that urinary amylase and urinary trypsinogen-2 are very sensitive and accurate indicators of AP, which is appropriate to identify the disease at an early stage, track its progression, and measure its severity. The high sensitivity level in diverse populations is a sign of its applicability as a screening technique, whereas moderate inconsistency in specificity is an indication of the persistence of confirmatory serum or imaging investigations in some instances. The urgent, non-invasive quality of urinary testing has practical benefits in an emergency and resource-constrained environment, which is consistent with the main aim of measuring urinary amylase as a diagnostic biomarker in AP.

Thirteen studies published between 2010 and 2025 were considered, and the sample sizes of the studies consisted of 51-458 participants. In diagnosing cases of AP, urinary amylase had a sensitivity of 62.9-97.3 and a specificity of 42.9-98.5. The values of the AUC found in selected studies ranged between 0.70 and 1.00, indicating good diagnostic performance. A number of studies, such as those of Judal et al. and Akula et al., showed the same or better diagnostic accuracy than that of serum amylase and lipase [9,13]. Its high NPVs (up to 98.3) also underscore the fact that it is a quick, non-invasive screening tool that can prove useful, especially in an emergency or resource-constrained environment.

**Figure 2:**
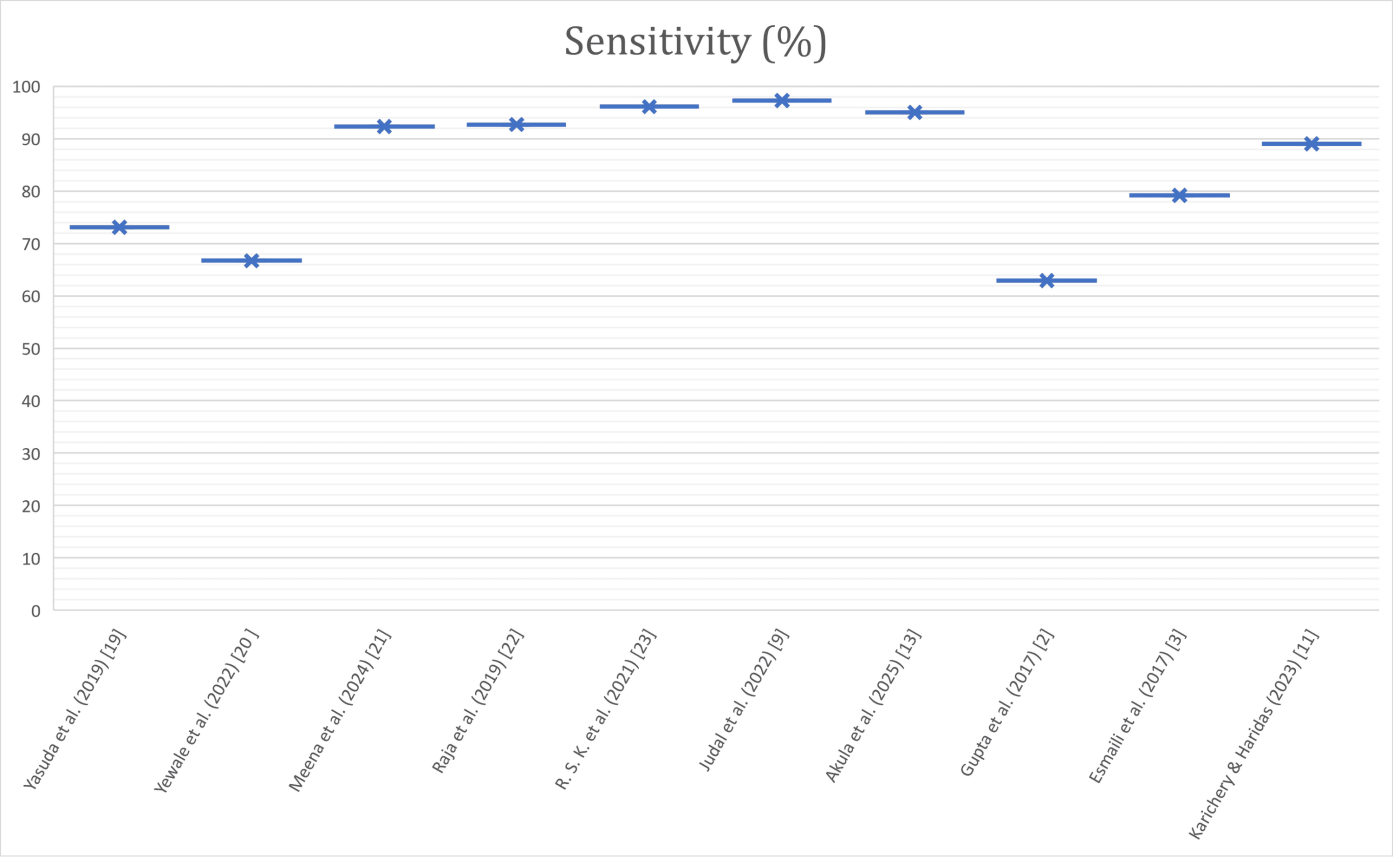
Comparison of urinary amylase in the included studies in terms of sensitivity (%) Sensitivity of urinary amylase for diagnosing acute pancreatitis across the included studies. Each point represents study-level sensitivity (%) extracted from the original report [2,3,9,11,13,19-23]. Where reported, values reflect admission or the earliest available sampling. No confidence intervals are shown when not provided. Higher values indicate better true-positive detection.

**Figure 3:**
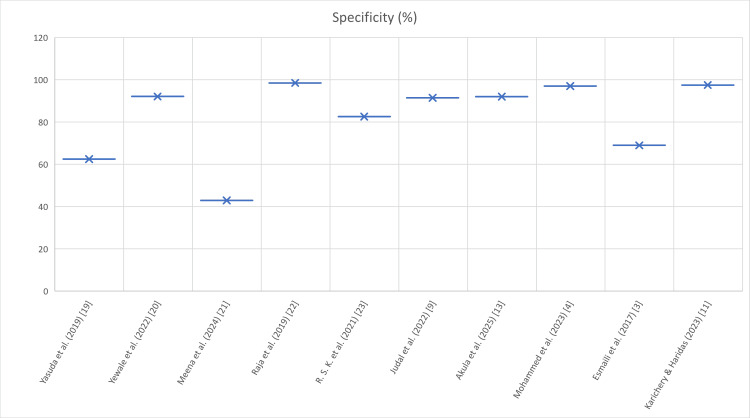
Comparison of urinary amylase in the included studies in terms of specificity (%) Study-level specificity (%) as reported. Variability reflects differing thresholds, timing of urine collection, and reference standards. While specificity varied among studies, several reported values above 80% [3,4,9,11,13,19-23].

**Figure 4:**
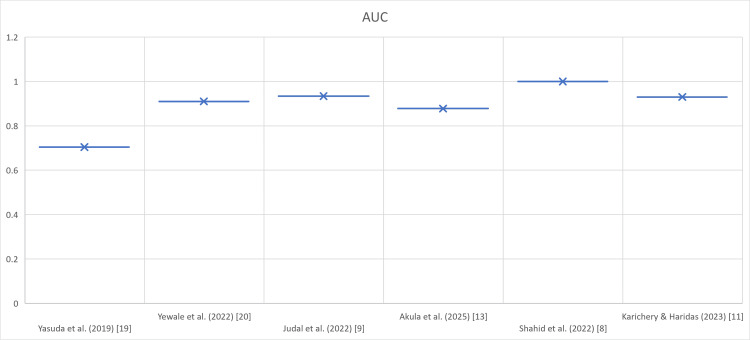
Comparison of urinary amylase in the included studies in terms of AUC AUC values (x-axis) reported in selected studies (y-axis) evaluating urinary amylase for acute pancreatitis diagnosis. The majority of studies showed AUC >0.85, suggesting a strong overall diagnostic performance [8,9,11,13,19,20]. AUC: area under the curve

Figures 2-4 depict the comparison of urinary amylase in the included studies in terms of sensitivity, specificity, and AUC.

Discussion

The systematic review assessed the diagnostic value of urinary amylase and urinary trypsinogen-2 in AP and summarized the results of 13 studies carried out in several countries. In general, the articles in the collection have constantly shown that urinary markers are very sensitive and clinical in the diagnosis of AP, with reported sensitivities of 62.9-100% and specificities of 42.9-100%. These results are consistent with the already existing published literature, indicating the use of urinary amylase as a non-invasive, rapid, and effective biomarker [7,8,13].

Traditionally, serum amylase and lipase are extensively utilized in AP diagnosis, but their clinical applicability is constrained by a short half-life, lower sensitivity, and reduced reliability in late and mild disease patients [1-3]. It is estimated that about 20-30% of AP can also have normal serum amylase at the onset, making it necessary to use other modes of diagnosis [4]. The literature reviewed in this paper affirms that urinary amylase is higher over a more extended time, which offers a useful benefit in the identification of AP in both early and late manifestations. As an example, Mogekar et al. proved that the level of urinary amylase was significantly higher than the level of serum amylase (mean 501 U/L vs. 311 U/L), and Akula et al. showed a sensitivity and a specificity of 95% and 92%, respectively, which points to the strengths of the marker in the clinical environment [7,13].

Urinary amylase had a comparable or better performance in a direct comparison with serum markers. Judal et al. had a sensitivity and specificity of 97.25% and 91.47%, respectively, almost identical to serum lipase (specificity 96.5%; AUC 0.945) [9]. Shahid et al. also affirmed the superiority of urinary amylase in diagnosing AP with an AUC of 1.0 [8]. Such results reveal the possibility of adding urinary amylase to the common medical practice, at least as a first-line or complementary test, as it is quicker, non-invasive, and easily repeatable to track the development of the disease.

Urinary amylase has a special clinical applicability in cases of emergency and limited resources, where the timely test is likely to lead to timely interventions and reduce unnecessary imaging or hospital waiting times [10,11]. Additionally, urinary biomarkers provide an advantage in severity stratification by correlating with disease severity, as demonstrated by Meena et al., who found that higher urinary trypsinogen-2 levels were associated with more severe disease. They may also offer additional clinical benefits, such as enabling the earlier initiation of fluid resuscitation based on urinary trypsinogen-2 results, thereby providing value beyond simple disease diagnosis [21]. 

In spite of these strengths, the heterogeneity between studies needs to be mentioned. Differences in assay procedures, cut-offs, patients, and study designs also led to a wide range of reported sensitivity and specificity. Although the majority of the studies faced low risks of bias (QUADAS-2) [17], some had uncertain or moderate risks concerning the little blinding or inconsistency in index test methods [4,21]. Such shortcomings demonstrate the necessity of standardized protocols of urinary amylase measurements and multicenter validation research on how to maximize clinical applications.

Future investigations must involve the determination of universal cut-off values, combined urine and serum assessment, and cost-efficacy and effect on patient outcomes. Moreover, the clinical use of urinary amylase could be further expanded through studies focusing on its role in early risk stratification and prognosis and assessment of treatment response, using early amylase measurements.

To summarize, this systematic review shows that urinary amylase and urinary trypsinogen-2 are the best biomarkers in the diagnosis of AP as they are non-invasive, highly sensitive, and reliable. Their use is similar or even better than that of traditional serum markers, especially in early, mild, or delayed presentations, and has viable benefits in emergency and resource-constrained settings. The cumulative amount of evidence suggests it is appropriate to integrate urinary amylase into mainstream clinical management practices, both as a diagnostic measure and as a possible disease monitoring tool, and thus increase the prompt disease detection and control of AP.

Limitations

Although the findings of this systematic review are rather strong, one must also take into consideration several limitations. To begin with, there was heterogeneity in the studies included in that the sample size, patient population, assay procedures, and cut-off values of urinary amylase and urinary trypsinogen-2 could have impacted the overall generalizability of the accumulated findings. Secondly, though the majority of the studies were deemed to be at a low risk of bias with QUADAS-2 [17], some of them were of unclear or moderate risk because of incomplete blinding, index test procedure variation, or lack of reporting on diagnostic performance metrics [4,21]. On the other hand, there were studies that only reported sensitivity or specificity, but not all the data on PPV or NPV and AUC, which does not allow conducting a fully quantitative meta-analysis. In the fourth place, most of the studies were based in one region (India); thus, this could expose external validity in other healthcare settings and ethnic groups. Lastly, variations in the time of urine sample collection when compared to the beginning of the symptoms were observed that may affect urinary amylase values and the accuracy of the diagnosis. The need to include such restrictions justifies the need to have standard guidelines, multicenter studies, and reporting of similarities of diagnostic measures to cement the evidence base and establish a clinical adoption of urinary amylase testing in AP.

## Conclusions

Sensitive, reliable, and clinically useful biomarkers of AP are urinary amylase and urinary trypsinogen-2, which have similar or even better diagnostic performance compared to traditional serum amylase and lipase. Incorporating urinary testing into routine clinical practice, either as a first-line or as an adjunctive test, can support earlier diagnosis, aid monitoring in later stages of disease, and help reduce the need for invasive tests. The optimal clinical use is suggested to be standardized measurement protocols and multicenter validation studies.
